# Activation of the Adipose Tissue NLRP3 Inflammasome Pathway in Cancer Cachexia

**DOI:** 10.3389/fimmu.2021.729182

**Published:** 2021-09-23

**Authors:** Joyce de Cassia Rosa de Jesus, Ariene Soares de Pinho Murari, Katrin Radloff, Ruan Carlos Macêdo de Moraes, Raquel Galvão Figuerêdo, Ana Flavia Marçal Pessoa, José César Rosa-Neto, Emídio Marques Matos-Neto, Paulo S. M. Alcântara, Flavio Tokeshi, Linda Ferreira Maximiano, Fang Chia Bin, Fernanda Bellotti Formiga, José P. Otoch, Marilia Seelaender

**Affiliations:** ^1^ Cancer Metabolism Research Group, Department of Surgery Laboratório de Investigação Médica (LIM26), Faculdade de Medicina, Universidade de São Paulo, São Paulo, Brazil; ^2^ Immunometabolism Laboratory, Institute of Biomedical Sciences, Universidade de São Paulo, São Paulo, Brazil; ^3^ University Hospital, Department of Surgical Clinic, Universidade de São Paulo, São Paulo, Brazil; ^4^ Department of Coloproctology, Santa Casa de São Paulo, São Paulo, Brazil

**Keywords:** neoplasms, cachexia, adipose tissue heterogeneity, inflammation, NLRP3 inflammasome

## Abstract

**Background:**

Cachexia is a paraneoplastic syndrome that accompanies and compromises cancer treatment, especially in advanced stages, affecting the metabolism and function of several organs. The adipose tissue is the first to respond to the presence of the tumor, contributing to the secretion of factors which drive the systemic inflammation, a hallmark of the syndrome. While inflammation is a defensive innate response, the control mechanisms have been reported to be disrupted in cachexia. On the other hand, little is known about the role of NLRP3 inflammasome in this scenario, a multiprotein complex involved in caspase-1 activation and the processing of the cytokines IL-1β and IL-18.

**Aim:**

based on the evidence from our previous study with a rodent model of cachexia, we examined the activation of the NLRP3 inflammasome pathway in two adipose tissue depots obtained from patients with colorectal cancer and compared with that another inflammatory pathway, NF-κB.

**Results:**

For CC we found opposite modulation in ScAT and PtAT for the gene expression of TLR4, Caspase-1 (cachectic group) and for NF-κB p50, NF-κB p65, IL-1β. CD36, expression was decreased in both depots while that of NLRP3 and IL-18 was higher in both tissues, as compared with controls and weight stable patients (WSC). Caspase-1 basal protein levels in the ScAT culture supernatant were higher in WSC and (weight stable patients) CC, when compared to controls. Basal ScAT explant culture medium IL-1β and IL-18 protein content in ScAT supernatant was decreased in the WSC and CC as compared to CTL explants.

**Conclusions:**

The results demonstrate heterogeneous responses in the activation of genes of the NLRP3 inflammasome pathway in the adipose tissue of patients with cancer cachexia, rendering this pathway a potential target for therapy aiming at decreasing chronic inflammation in cancer.

## Introduction

Cancer is among the four major causes of premature death in the world. The risk of getting sick and dying from cancer is related to hereditary aspects, increased life expectancy, the process of aging, lifestyle habits, the influence of risk factors, and the environment in which people live in. In low- and middle-income countries, such as Brazil, there has been a reduction in the number of cases of infectious agents-related cancers, such as those in the oral cavity and cervix, and an increase in the types that are more common in developed countries, including colorectal cancer (CRC). The latter is the third most incident type of cancer in the world (1).

Cancer cachexia is an overly debilitating and harmful paraneoplastic syndrome that compromises the therapeutic response and quality of life of patients. Its etiology is complex and not yet fully understood, but sustained systemic inflammation is a peculiar feature, stimulating exacerbated energy expenditure through cytokine secretion by the tumor and host tissues in response to the tumor, causing muscle and adipose tissue loss (2–7).

Considered a multiorgan, white adipose tissue performs an important role in health and disease. It is an active endocrine organ, responsible for the secretion of steroid precursors of hormones, cytokines, autacoids, fatty acids, and derivatives, and the so called adipokines, with functions involved in the regulation of fat storage, energy metabolism, food intake, insulin sensitivity and immunity, amongst many other (8–14). Under the presence of sustained systemic inflammation observed in cancer cachexia, the metabolism and function of the adipose tissue are impaired, in association with morphological and molecular changes (15). Studies have shown that the subcutaneous adipose tissue is the first to be affected in cancer cachexia, but the inflammatory mechanisms that cause adipose mass depletion and increased tissue inflammatory output remain not fully elucidated (15–18).

Adipose tissue acts at the crossroads between metabolism and immunity: as it is an endocrine organ, it is capable of regulating the immune system, and the signaling between these two compartments displays a delicate and intricate balance (2–4, 7). Inflammation is a protective innate response to danger signals and in the presence of cancer cachexia, diverse inflammatory pathways are activated, perhaps in an attempt to restore homeostatic balance (5). Inflammasomes are key regulators of inflammatory processes and are implicated in diseases such as gout and colorectal cancer (6, 8–10). NLRP3 inflammasome is a multiprotein complex responsible for the maturation of caspase-1 and the processing of cytokines IL-1β and IL-18 (11–14). It is considered the most versatile of this family of intracytoplasmic receptors, owing to its activation by a wide range of factors (15–17).

We have previously shown that there is a distinct pattern of secretion of inflammatory factors by the tumor and the adipose tissue (subcutaneous and peritumoral pads) and differences in the tumor microenvironment in cachectic patients versus weight-stable patients (18, 19). In a previous study we showed, for the first time, that the inflammasome pathway may be involved in adipose tissue inflammation in a rodent model of cancer cachexia (20).

Due to the contribution of adipose tissue to several biological processes and to cancer cachexia, we sought to investigate the activation of components of the NLRP3 inflammasome pathway in the two white adipose tissue depots in patients with colorectal cancer. A PubMed search retrieves only 5 articles under “inflammasome and cachexia”. As demonstrated for obesity, different adipose compartments present heterogeneous function/contribution in health and disease. The present study was designed as to confirm that the changes reported previously for animal model of cachexia (20), with induction of the inflammasome pathway, are also present in the human form of the disease.

## Methods

### Patients, Ethics and Cachexia Diagnosis

This research project was approved, following the Declaration of Helsinki, by the Research Ethics Committees of the “Faculdade de Medicina” (CAAE no. 54503716.1.0000.0065), of the University Hospital (CAAE no. 54503716.1.3001.0076) and the Institute of Biomedical Sciences (CAAE no. 54503716.1.3002.5467) of the University of São Paulo (USP). Twenty-eight volunteers were recruited into the study at diagnosis. Informed consent before to surgery was obtained from all participants. Inclusion criteria were diagnosis of colorectal cancer with an indication for surgery, or with a surgical indication for correction of abdominal wall hernias (umbilical and inguinal), cholecystectomies, and abdominal proctoplexy, and willingness to participate in the study. Exclusion criteria: having received antibiotic therapy, anti-inflammatory therapy, or anti-cancer treatment. extreme low weight or obesity (BMI <18 and >30 kg/m², respectively), chronic heart failure, liver or kidney failure, chronic obstructive pulmonary disease, acquired immunodeficiency syndrome (AIDS), inflammatory bowel disease (IBD), hepatitis and autoimmune diseases.

For the diagnosis of cachexia, the criteria established in international consensus by Evans and others were employed (21): unintentional weight loss in the last 12 months or less or body mass index less than 20 kg/m²; decreased muscle strength; fatigue; anorexia; altered biochemical parameters (C-Reactive Protein (CRP) >5 mg/l; Hemoglobin <12 g/dl; albumin < 3.5 g/dl). Subjects were distributed into 3 groups: control patients (CTL, n = 8), weight-stable colorectal cancer patients (WSC, n=10), and cachectic colorectal cancer patients (CC, n = 10).

### Obtention of Biopsies

During the surgical procedure, fragments of approximately 1g of subcutaneous adipose tissue (ScAT) and visceral adipose tissue (mesenteric) immediately close to the tumor (peritumoral adipose tissue, PtAT) were collected.

### Explant Incubation

Adipose tissue explants were distributed in 12-well culture plates, with approximately 500 mg per well and incubated in DMEM containing 10% fetal bovine serum (FBS), supplemented or not with lipopolysaccharide (LPS, from Escherichia coli serotype O55:B55, SIGMA®, product number L2880) (1 µg/ml), in a humidified atmosphere of 10% CO2 at 37°C for 24h (22, 23). The unstimulated well received 500 µl DMEM, and the stimulated well received 490 µl DMEM and 10 µl LPS. After 24 hours, the culture medium was collected for protein quantification of IL-1β, IL-18, and caspase-1, and the explants were frozen at -80°C for further RNA extraction.

### RT-PCR

Total RNA of subcutaneous and peritumoral adipose tissue was extracted with Trizol^©^ reagent (Invitrogen, Carlsbad, CA) observing the manufacturer’s instructions. The concentration of total RNA was evaluated with the spectrophotometer. The cDNA was obtained by reverse transcription (RT) using 1µg of total RNA from each sample per reaction, in a final volume of 20 µl. The target gene was normalized to the reference gene GAPDH. The mRNA levels were determined using the 2^-ΔΔCt^ method, in which, for each sample, the ΔCt value was obtained by subtracting the control gene values from those of the target gene; then, the average ΔCt value of the control group was subtracted from the sample to get a -ΔΔCt value (24). The primers used are listed in **Table 1**.

**Table 1:** List of primers used.

Gene (specie)	Sequence 5’→3’
TLR4, mRNA NM_138554.4 *Homo sapiens*	Forward primer: TTT ATC CAG GTG TGA AAT CCA G
	Reverse primer: AGA TGC TAG ATT TGT CTC CAC AG
CD36, mRNA NM_000072.3 *Homo sapiens*	Forward primer: GGA AGA ACA AAT CTA TAC ACA GGG
	Reverse primer: GAT TAA TGG TAC AGA TGC AGC CT
NF-κB p50, mRNA NM_003998.3 *Homo sapiens*	Forward primer: ACA CTT AGC AAT CAT CCA CC
	Reverse primer: AAT CCT CCA CCA CAT CTT CC
NF-κB p65, mRNA NM_021975.3 *Homo sapiens*	Forward primer: ATA CCA CCA AGA CCC ACC C
	Reverse primer: GCC TCA TAG AAG CCA TCC
NLRP3, mRNA NM_004895.4 *Homo sapiens*	Forward primer: CAT TGA GAA CTG TCA TCG GG
	Reverse primer: GCT GTT CAC CAA TCC ATG AG
Caspase-1, mRNA NM_033292.3 *Homo sapiens*	Forward primer: TGC TTC GGA CAT GAC TAC AG
	Reverse primer: GTT TCT TCC CAC AAA TGC CT
IL-1β, mRNA NM_000576.2 *Homo sapiens*	Forward primer: ACA GAT GAA GTG CTC CTT CC
	Reverse primer: ATC TTC CTC AGC TTG TCC A
IL-18, mRNA NM_001562.3 *Homo sapiens*	Forward primer: CCT GGA ATC AGA TTA CTT TGG C
	Reverse primer: GGG TGC ATT ATC TCT ACA GTC
GAPDH, mRNA NM_002046.6 *Homo sapiens*	Forward primer: CCT CTG ACT TCA ACA GCG AC
	Reverse primer: CGT TGT CAT ACC AGG AAA TGA G

TLR4, Toll-like receptor 4; CD36, Cluster of Differentiation 36; NF-κB p50, Nuclear Factor kB, p50 subunit, NF-κB p65; Nuclear Factor kB, p65 subunit; NLRP3, NLR, Family pyrin domain containing 3; IL-1β, Interleukin-1 beta; IL-18, Interleukin-18; GAPDH, Glyceraldehyde-3-phosphate dehydrogenase.

### Protein Expression Assessment

The protein concentration of the culture medium supernatant was determined by the Bradford method (Bradford Protein Assay, Bio-Rad Laboratories). Afterward, the quantification of proteins (caspase-1, IL-1β, and IL-18) was performed using ELISA. The kits used for these tests were Human Caspase-1 SimpleStep ELISA^®^ Kit (Abcam, Cat. No. ab219633), ELISA Max™ Deluxe Set Human IL-1β (Biolegend^®^, Cat. No. 437004), and RayBio^®^ Human IL-18 ELISA Kit (RayBiotech, Cat. No. ELH-IL18).

### Statistical Analysis

Comparisons among groups were carried out using the ANOVA 1-way test, for data with normal distribution, or the Kruskal-Wallis test, for data with asymmetric distribution. To assess the dispersion of nominal variables, the Chi-square test (*X* ^2^) was used. For multiple comparisons, the ANOVA 2-way test was used. The Uncorrected Fisher’s LSD post-test was applied. Outliers were identified using the Rout method and removed from the analysis.

For all analyses, a significance level of p<0.05 was adopted, using the Prism^®^ version 8.0 statistical software (GraphPad Software, Inc.). Data were expressed as mean ± standard error unless otherwise indicated.

## Results

### Clinical Characteristics of Patients

**Table 2** shows the clinical characteristics of the patients. Age, sex, and BMI were similar among groups. Height was lower in the CC versus CTL. Weight loss was greater in the CC. The comorbidities reported at the time of recruitment by the volunteers were allergy, dyslipidemia, hypertension, asthma, hypothyroidism, hypercholesterolemia, depression, epilepsy, type 2 diabetes, pre-diabetes, and pain.

**Table 2:** General and clinical characteristics.

	CTL	WSC	CC	p
**n**	8	10	10	
**Women (n) (%)**	3 (38%)	6 (60%)	9 (90%)	0.065
**Men (n) (%)**	5 (62%)	4 (40%)	1 (10%)	
**Age (years)**	49.5 ± 5.7	57 ± 3.2	64.5 ± 5.8	0.13
**Height (m)**	1.69 ± 0.03	1.60 ± 0.02^a^	1,59 ± 0.03^b^	0.02*
**Previous weight (kg)**	68.8 ± 4.8	65.6 ± 4.6	68.3 ± 3.6	0.85
**Current weight (kg)**	68.5 ± 4.7	61.6 ± 4.0	60 ± 2.5	0.27
**Weight loss (%)**	0 [0; 0,9]	4.1 [0; 9.2]	10.5 [7.9; 17.1] ^b^	0.01*
**BMI (kg/m²)**	23.8 ± 1.3	23.7 ± 1.2	23.8 ± 0.8	0.99
**Tumor site**				
** Right colon (n) (%)**	N. A.	2 (20%)	4 (40%)	0.62
** Left colon (n) (%)**	N. A.	8 (80%)	6 (60%)	
**Tumor staging (TNM)**				
** I**	N. A.	1	1	0.8
** II**	N. A.	5	3	
** III**	N. A.	3	4	
** IV**	N. A.	1	2	
**Comorbidities**				
** Allergy**	1	N. A.	N. A.	NA
** Dyslipidemia**	N. A.	2	N. A.	
** Hypertension**	N. A.	2	4	
** Asthma**	N. A.	N. A.	1	
** Hypothyroidism**	N. A.	N. A.	2	
** Hypercholesterolemia**	N. A.	N. A.	1	
** Depression**	N. A.	1	1	
** Epilepsy**	N. A.	1	N. A.	
** Type 2 Diabetes**	N. A.	N. A.	1	
** Pre-diabetes**	N. A.	N. A.	1	
** Pain**	N. A.	N. A.	1	

Data are presented as mean ± S.E.M. or as median [first quartile; third quartile]. ^a^ Significant difference (WSC versus CTL). ^b^ Significant difference (CC versus CTL). *p < 0.05. BMI, body mass index; TNM, tumor-node-metastasis (Classification of Malignant Tumors). N.A., not applied.

We grouped the colorectal tumors according to location, in the right colon (cecum, ascending, hepatic flexure, and proximal 2/3 of the transverse colon) and left colon (1/3 distal of the transverse colon, flexure splenic, descending, sigmoid, rectosigmoid, and rectum). As for the anatomical location of the tumor, most patients presented cancer in the left colon, both for WSC group (80%) and CC (60%). Regarding tumor staging, the percentages were higher in stages II and III, in both groups with cancer.

There was no difference between the mean age or sex among the groups. Height was lower in the CC group, and the two cancer groups were equal in terms of BMI. BMI calculation is not an adequate tool to assess body composition, but it has been used as a cachexia marker (25). Analyzing several meta-analyses, the CUP Panel (Continuous Update Project, World Cancer Research Fund) indicated that individuals with BMI > 27 kg/m^2^ are at a higher risk of developing CRC (26). About 80% of cancer patients experience involuntary weight loss, a cardinal clinical criterion in the diagnosis of cachexia. In this study, the usual weight of CC was equal to that of CTL at the time of recruitment. The average weight loss percentage of WSC was 5.1%, and in the CC group it was 11.6%, in 12 months or less.

Three patients in WSC group and nine in CC group reported having comorbidities; 2 from the WSC group and 3 from the CC group reported having more than one comorbidity. The most frequent comorbidity was systemic arterial hypertension. Colorectal cancer usually presents in a greater proportion in elderly patients, who also tend to have more chronic diseases.

Dysregulated lipid metabolism is also a hallmark of cancer, as it has the crucial function of providing constituents for the cell membrane and tumorigenic pathways (27). The levels of total cholesterol and the LDL fraction were higher in WSC when compared to CTL; these differences were not observed when comparing CC to the control (non-cancer) group. The post-test analysis showed the levels of total cholesterol and LDL fraction to be lower in CC when compared to WSC. The HDL fraction, serum triglycerides and glucose did not vary among the groups. For the diagnosis of cachexia, we used serum hemoglobin, CRP, and albumin (21). Serum hemoglobin levels were lower in the CC compared to the CTL, indicating anemia in cachectic patients. CRP levels were higher in CC in relation to the CTL, showing systemic inflammation in CC group. Albumin showed no difference among groups (**Table 3**).

**Table 3:** Biochemical parameters.

	CTL	WSC	CC	p
**n**	8	10	10	
**Total cholesterol (mg/dL)**	201.6 ± 13.0	246.9 ± 17.6^a^	165.2 ± 5.7^b^	0.0005***
**LDL cholesterol (mg/dL)**	119.5 ± 15.2	164.4 ± 14.3^a^	108.6 ± 9.5^b^	0.01*
**HDL cholesterol (mg/dL)**	51.9 ± 4.5	53.2 ± 4.2	46.5 ± 5.9	0.5
**Triglycerides (mg/dL)**	115.9 ± 25.2	116.1 ± 17	113.6 ± 11.5	0.9
**Glucose (mg/dL)**	98.9 ± 2.3	98.6 ± 7.5	100.4 ± 3.9	0.9
**Hemoglobin (g/dL)**	14.2 ± 0.8	13.3 ± 0.5	10.7 ± 0.6^b^	0.001
**C Reactive Protein (mg/L)**	3.8 ± 1.7	5.6 ± 1.7	9.6 ± 1.7^b^	0.02
**Albumin (g/dL)**	4.6 ± 0.1	4.3 ± 0.3	4.1 ± 0.1	0.2

Data are expressed as mean ± S.E.M. ^a^ Significant difference (WSC versus CTL). ^b^ Significant difference (CC versus WSC). *p < 0.05, ***p < 0.005.

### Analysis of Gene and Protein Expression

**Figure 1:**
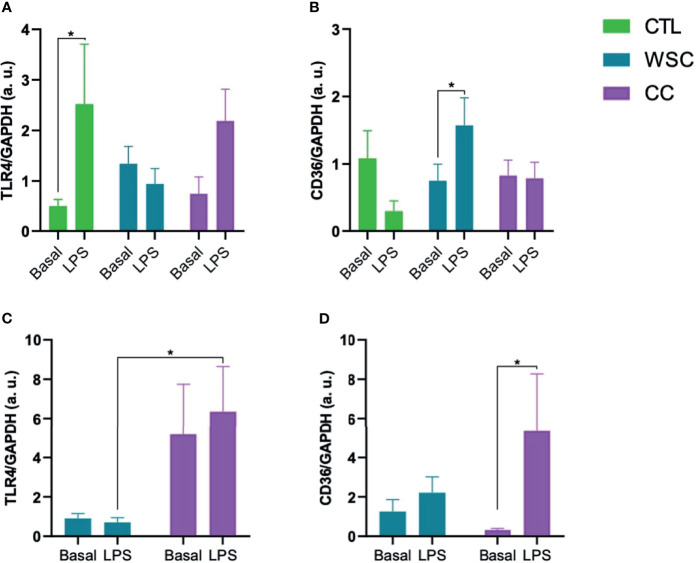
Gene expression of receptors involved in inflammasome activation. **(A, B)** TLR4 and CD36 gene expression in ScAT and **(C, D)** TLR4 and CD36 gene expression in PtAT. TLR4, Toll-like receptor 4; CD36, Cluster of Differentiation 36; GAPDH, Glyceraldehyde-3-phosphate dehydrogenase a.u.; arbitrary units; LPS, lipopolysaccharide. *p < 0.05.

Two important receptors in the adipose tissue that are relevant for inflammasome activation are TLR4 and CD36. The analysis of the unstimulated gene expression of TLR4 showed no distinct differences among the study groups in subcutaneous or peritumoral adipose tissue explants. Incubation with LPS induced TLR4 in ScAT explants in CTL and CC, but in the latter, significance was not reached. Basal CD36 gene expression did not differ among the explants obtained from the 3 groups in either of the adipose tissue depots. In subcutaneous explants from WSC, CD36 expression increased in response to LPS, as well as in the peritumoral tissue from CC (**Figure 1**).

**Figure 2:**
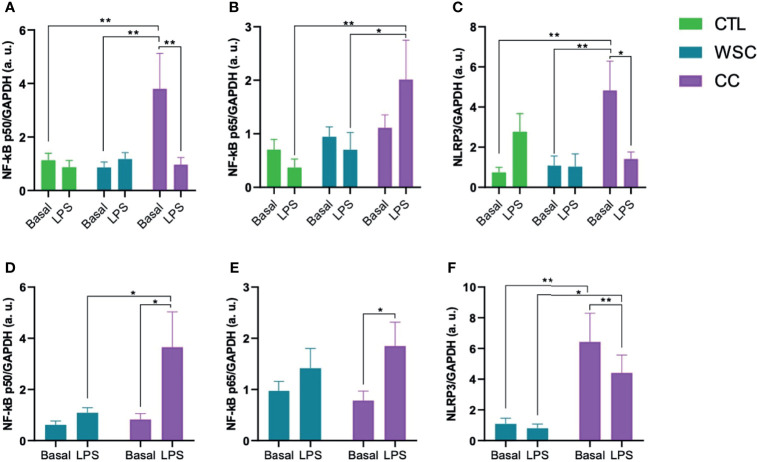
NLRP3 induction via NF-kB Pathway. **(A–C)** NF-κB p50/p65 and NLRP3 gene expression in ScAT and **(D–F)** NF-κB p50/p65 and NLRP3 gene expression in PtAT. NF-κB p50: Nuclear Factor kB, p50 subunit; NF-κB p65: Nuclear Factor kB, p65 subunit; NLRP3, NLR, Family pyrin domain containing 3; GAPDH, Glyceraldehyde-3-phosphate dehydrogenase; a.u., arbitrary units; LPS, lipopolysaccharide. *p < 0.05 and **p < 0.01.

The activation of the TLR4, as well as the CD36 receptor cascade, result in the release of the NF-κB p50/p65 complex from IκB and its translocation into the nucleus, where the transcription of NLRP3 is induced. The gene expression analysis of the NF-κB subunits p50 and p65 revealed that the mean p50 expression in subcutaneous CC explants was about 2-fold higher than in CTL or WSC. LPS stimulus suppressed p50 expression in CC subcutaneous explants, while in the peritumoral adipose tissue an opposite effect of LPS led to an increase in p50 expression in CC. No differences in basal NF-κB p65 gene expression was observed among the groups for any of the studied adipose depots. In contrast to p50, p65 gene expression increased in response to incubation with LPS in CC subcutaneous explants. The same effect of LPS on p65 was observed in peritumoral adipose tissue explants from CC group. The unstimulated mean NLRP3 gene expression in ScAT explants was about 5-fold higher than in CTL or WSC. This was also the case for PtAT. LPS displayed a suppressive effect on NLRP3 expression in CC in both studied adipose tissue depots. With a decrease of approximately 60%, this effect was stronger in ScAT than in PtAT, where NLRP3 expression was reduced by a third (**Figure 2**).

**Figure 3:**
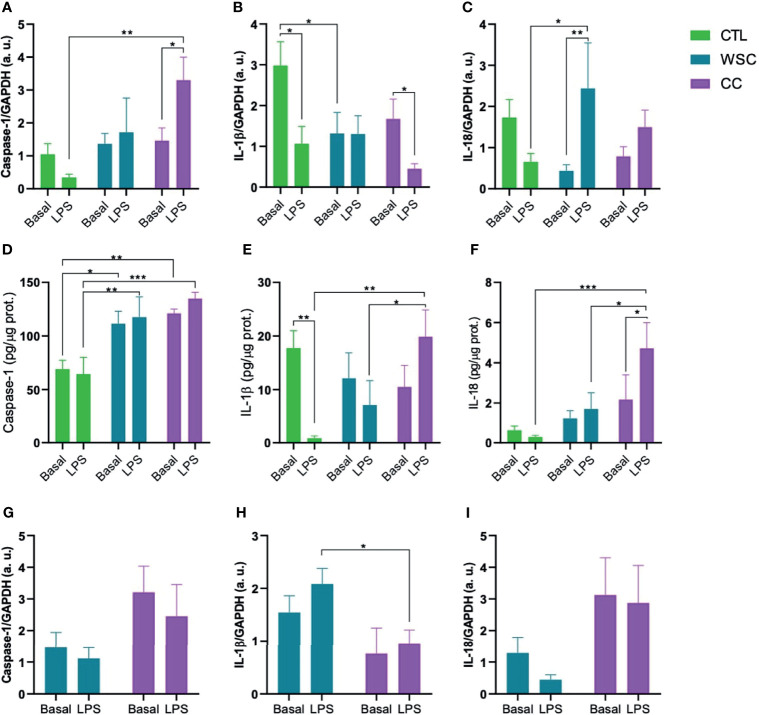
Inflammasomal cytokine activation mediated by caspase-1. Gene expression **(A–C)** and protein levels **(D–F)** of caspase-1, IL-1β and IL-18 in ScAT and **(G–I)** genes expression of caspase-1, IL-1β and IL-18 in PtAT. IL-1β, Interleukin-1 beta; IL-18, Interleukin-18; GAPDH, Glyceraldehyde-3-phosphate dehydrogenase; a.u., arbitrary units; LPS, lipopolysaccharide. *p < 0.05; **p < 0.01 and ***p < 0.005.

The inflammasome activates caspase-1, which in turn, activates the cytokines IL-1β and IL-18 by cleaving the pro-peptide. In the subcutaneous adipose tissue explants, basal caspase-1 gene expression was not different among the groups. However, caspase-1 protein levels in the explant culture medium were significantly higher in ScAT of WSC and CC, as compared to CTL. LPS increased caspase-1 gene expression in CC ScAT explants, but this LPS effect was not observed when assessing the protein concentrations in the explant incubation supernatant. Although there was no difference in Caspase-1 gene expression between WSC and CC in ScAT, basal caspase-1 expression in PtAT of CC patients was higher than in the tissue from WSC. Unlike the ScAT of CC, the peritumoral adipose tissue of CC was not sensitive to the LPS stimulus. Basal IL-1β gene expression was lower in both cancer groups when compared to CTL, with a significant difference in WSC and a trend for CC. In peritumoral adipose tissue there was also a trend for a lower IL-1b expression in CC compared to WSC. However, the basal IL-1β protein content in ScAT explant supernatant was not significantly different among the groups. Incubation with LPS suppressed IL-1β gene expression in ScAT of CTL and CC, while there was no LPS-induced effect on IL-1β gene expression in PtAT. The downregulating effect of LPS on IL-1β in ScAT CTL explants was also confirmed by the lower protein levels found in the supernatant. Interestingly, in the supernatants obtained from CC explants, the LPS incubation caused an increase in IL-1b protein content, which was contrary to the gene expression data. Basal IL-18 gene and protein expression did not differ among the groups for ScAT explants, whereas in PtAT tissue IL-18 gene expression tended to be higher in CC when compared to WSC. In ScAT explants, incubation with LPS resulted in an increase of IL-18 gene expression in WSC which was not reflected by the supernatant protein data in this group, while in CC, the challenge led to a significant increase in IL-18 protein content. No effect of LPS was observed in the peritumoral depot (**Figure 3**).

## Discussion

### Clinical Findings

An increase in the number of cases of CRC has been observed in patients under the age of 50 years, mainly in the left colon. There are embryological, biological, anatomical, and molecular differences in CRC located on the right and left sides (28), being considered as different biological entities (29). Tumors located in the right colon are associated with anemia, advanced staging, and advanced age; the lesions tend to be polypoid and bulky and grow into the lumen of the organ, while the characteristics of left-sided tumors are infiltrating and constrictive lesions causing obstruction (29, 30). One study demonstrated that patients with left-sided CRC had longer disease-free survival than those with right-sided, depending on the stage of the tumor. They also showed that, among patients who lost weight, the highest percentage was among patients with CRC on the right side (31). In our study, most tumors were concentrated in the left side of the colon (80% in WSC and 60% in CC). Lim and coll. reported that patients with tumors on the right side of the colon presented increased tumor size, advanced cancer, and lower disease-free and overall survival rates (30). Our work shows the highest percentages at stages II and III. Even if they have the same type of cancer and tumor burden, one patient may develop cachexia and another may not; Fearon and others attributed this fact to possible genotypic variations in immunity (32). We (18, 19) have previously shown that there is also a distinct pattern of secretion of inflammatory factors by the tumor and differences in the tumor microenvironment in cachectic patients versus weight-stable patients.

Cancer cachexia is a syndrome that is difficult to diagnose and treat; its etiology remains partly unknown, and its pathophysiology is complex and poorly understood, despite the efforts of decades of study. It is difficult to disentangle its roots from cancer and it is common to have it confused with other conditions, such that, in most cases, cachexia is only diagnosed when there is no longer a turning point in the patient’s response, that is, refractory cachexia. Therapeutic strategies currently used for cancer cachexia include exercise, nutritional support and surgical removal of the tumor (especially of obstructive malignancy) (33). Clinical trials employ protocols of monodrug or a combination of drugs and nutrients (34–36). However, so far, no therapy has been described to comprehensively ameliorate cachexia.

Cachexia not only impacts cancer progression but also compromises the therapeutic response, increases treatment costs and length of hospitalization (37), and is the immediate cause of death in at least 20% of patients (33, 38). In a systematic review, Anker and others estimated that, of patients diagnosed with colorectal cancer, 50% are at risk of developing cachexia and calculated the 5-year survival rate for patients with both pathologies at 66% (39).

The aim of the study was to confirm in human cachexia what we have previously shown in animal models of the disease: white adipose tissue plays a relevant role for sustained systemic inflammation. In these earlier studies we have reported that adipose tissue response in cachexia was not uniform in terms of tissue architectural changes, nor did different anatomical depots contribute to the same extent to inflammation (7, 19, 20, 40–48). Furthermore, while the NF-κB pathway has been accredited much importance in cachexia, the inflammasome pathway contribution remains unclear. Thus, we compared the activation of both intracellular pathways in human tissue, as to investigate whether there could be differences in comparison to models (with which most molecular studies are carried out).

When assessing whether there was heterogeneity in the inflammatory response of adipose tissue depending on the anatomical location (mesenteric, retroperitoneal, and epididymal) in an animal model of cachexia, Neves and others (20) reported a marked heterogeneous response having demonstrated that the inflammasome pathway was involved in time-dependent adipose tissue inflammation in cachectic animal adipose tissue, and that adipocytes obtained from different depots presented heterogeneity in the response.

ScAT and VAT (corresponding to PtAT, in the present study scenario) are very distinct, so differences in gene and protein expression profiles may contribute to the observed disturbances. Different fat depots differ in cell size, physiological function, and contribution to disease states (49). In VAT, adipocytes have greater lipolytic activity and generally secrete more pro-inflammatory cytokines than ScAT, contributing to systemic inflammation associated with metabolic disorders. In ScAT, the storage of triacylglycerols occurs over the long term, while lipolysis is lower, being this a more, more densely vascularized, and metabolically less active than VAT. Finally, this tissue shows lower density of secretion of pro-inflammatory factors in humans (50).

Zoico and colleagues described *in vitro* PtAT morphofunctional changes, for example, shrinkage in size and number of adipocytes with intense lipolysis and a higher rate of macrophage infiltration (51); the same was seen in subcutaneous adipose tissue of cachectic patients (47). To address the importance of different intracellular pathways in the two adipose depots we studied under basal or stimulated conditions, explants of subcutaneous and visceral white adipose tissue.

LPS was added to the incubation medium of explants as a stimulus to induce the entire NLRP3 inflammasome signaling cascade. Yet, by addressing the unstimulated expression in the subcutaneous and peritumoral tissues, reflecting the actual condition of patients at the time of surgery, we already managed to identify differences. It has been shown that adipose tissue expresses all TLR subtypes, which is associated with NF-κB activation and subsequent release of cytokines (52, 53). TLR4 mRNA showed a different basal expression pattern in ScAT and PtAT explants. While in ScAT, unstimulated TLR4 gene expression was not altered between the study groups (**Figure 1**), in PtAT, TLR4 gene expression was higher in CC than in WSC explants. Interestingly, both adipose tissue depots also differed in their sensitivity towards LPS. In the ScAT explants of CTL and CC TLR4 expression was upregulated by LPS, while in CC explants derived from the PtAT depot LPS incubation failed to induce TLR4. It is possible that CC PtAT TLR4 expression reached already a status of saturation. On the other hand, as the expression was found to be already high, one may suggest that no further increase, as induced by LPS could be achieved. This might be also a possibility for explants of the WSC group, since neither ScAT, nor PtAT explants showed further TLR4 induction by LPS. In the literature, it was shown, that the GTPase Rab7, which is currently gaining importance as an oncosuppressor (54), impairs TLR4 by promoting its lysosomal degradation (54) and may therefore play a role in immune-suppressive mechanisms during cancer progression and cachexia development.

CD36 is not directly part of the NLRP3 inflammasome pathway but is an important signaling molecule interacting with TLR4 (55). CD36 (also known as fatty acid translocase) is an integral membrane glycoprotein expressed in most cells, particularly those involved in glucose homeostasis, such as adipocytes and skeletal muscle cells, facilitating the uptake of long-chain fatty acids and anionic or oxidized lipoproteins (56, 57). CD36 gene signaling has been implicated in the development of chronic diseases (55) and genetic variants have been associated with lipid abnormalities and susceptibility to metabolic syndrome in humans (58). Although in both adipose tissue depots CD36 gene expression was not altered in the studied groups, it was induced by LPS only in WSC ScAT explants and CC PtAT explants. This is in contrast to the results of TLR4 in CC PtAT explants and could point to shifts between different inflammasome activation routes due to tissue-specific changes during cancer cachexia progression.

The activation of TLR4 or CD36 results in the activation of NF-κB, which is a family of five transcriptional factor subunits, that are activated canonically (which acts on inflammation) and non-canonical (which regulates various immune functions), responding to different types of receptors and their ligands. Control the expression of several genes involved in inflammation, immunity, cell survival, proliferation, and differentiation (59–65).

The gene expression of the two NF-κB domains was not significantly different (**Figure 2**) in the adipose tissue of the patients. However, the activity of NF-κB depends on posttranslational modifications such as the cleavage of the IκB, and previous analysis of our group already showed changes in NF-κB complex proteins in adipose tissue in cancer cachexia (62). In both ScAT and PtAT CC explants, p65 was upregulated by LPS. NF-κB p50 followed that pattern in CC PtAT explants but in CC ScAT explants LPS had a contrary effect on p50 and lead to downregulation.

The expression of NLRP3 was increased in the cachectic group in both tissues of patients in the CC group. Experimental animal studies have linked NLRP3 inflammasome activation to inflammatory bowel diseases (15, 16). In IL-10 gene knock-out animals, the absence of this molecule led to uninterrupted activation of the inflammasome, due to the constant stimulation of microorganisms, causing chronic colitis (16). Knock-out animals for the nlrp3 gene exhibited greater weight loss (66). Studies on single nucleotide polymorphisms in the nlrp3 gene demonstrate susceptibility to Crohn’s disease, a risk factor for CRC (67), suggesting that a defective innate immune response impairs the removal of antigens and pathogens, leading to disease development (68). High level of NLRP3 expression correlated with advanced tumors, occurrence of metastases, vascular and lymph node invasion in patients with CRC (69). Deficiency in the genes nlrp3 and caspase-1 is related with a strong inflammatory response and destruction of the epithelial barrier, with consequent dysplasia, tumorigenesis, and greater tumor burden in the colon (70, 71). Caspase-1 gene knock-outs show weight loss greater than 20% of initial weight, drastic colonic shortening, and more severe signs of epithelial injury than control animals, indicating that caspase-1 is necessary for tissue regeneration and repair after damage (72). Although basal Caspase-1 gene expression showed no alterations among the groups for ScAT, the protein levels in the explant culture supernatant were higher in WSC and CC when compared to the CTL group. It remains unclear whether the active form of Caspase-1 is also increased, as we did not evaluate this parameter. In PtAT Caspase-1 gene expression tended to be higher in CC than in WSC explants.

Gene expression for the cytokine IL-1β in the CC was increased in ScAT and decreased in PtAT, as previously demonstrated by our group by Riccardi et al (73). Through the action of the inflammasome, Caspase-1 is activated resulting in the cleavage and activation of IL-1β and IL-18. High concentration of IL-1β and IL-18 cytokines is observed in patients with Crohn’s disease (74). The unregulated expression of IL-1β has been associated with inflammation of the intestinal mucosa in patients with IBD, *in vitro* and in animal models, as it can promote the epithelial-mesenchymal transition in intestinal cells (15, 75), with loss of expression of e-cadherin, thus contributing to the invasion, growth, and metastasis (75). Stage II patients who presented polymorphisms for the IL-1β gene had a higher risk of CRC recurrence (76). Basal ScAT explant culture IL-1β protein content and mRNA expression appeared to be lower in WSC and CC (**Figure 3**). The experimental design does not allow a conclusion about the activation of IL-1β. However, the increase in IL-1β protein content after LPS incubation points to a higher capacity for cytokine production in ScAT in CC under immune challenging conditions. Therefore, we postulate that besides involvement in tumor progression, this cytokine, deriving from the ScAT contributes to cachexia in patients.

IL-18 is constitutively expressed in some tissues, such as the intestine, where it contributes to the integrity of the intestinal barrier and the maintenance of a healthy microbiota (77). Gene and circulating expression of IL-18 increase in gastrointestinal cancers, with dual activity in CRC: in early stages, it promotes tissue repair protection, by down-regulating the IL-22 binding protein (IL-22BP); IL-22BP controls the biological activity of IL-22, which protects intestinal cells from damage during inflammation; and later, IL-18 acts by stimulating tumor growth and cell invasion (15, 78). IL-18 protein levels in ScAT supernatant were also decreased in the WSC and CC group and showed a similar sensitivity to LPS though with lower fold change. In PtAT IL-1β and IL-18 mRNA showed an opposite expression pattern as IL-1β mRNA expression was higher in WSC than in CC and Il-18 lower in WSC than in CC. Protein analysis in PtAT would help to draw a more distinct picture of adipose tissue depot-specific production of inflammasomal targets.

The present results confirm the importance of the adipose tissue as a contributor to inflammation in human cachexia, showing that the regulation of both NF-κB and inflammasome pathway are depot-specific, paving avenues for targeted treatment.

This study had several limitations. The volunteers’ previous body weight was reported by the patient and, therefore, inaccuracies are possible. It was not possible to perform the analysis of the patients’ body composition using DEXA because there was not enough time between recruitment and surgery. The number of individuals in each group was low due to the difficulty of obtaining surgical paired samples of subcutaneous and peritumoral adipose tissue. Due to the intrinsic variation between samples of human tissue, some analyzes were not performed for all individuals initially enrolled, as some samples showed aberrant values. It was also not possible to assess the protein profile in the PtAT, with biopsies being of small dimensions. Since the primary intent of our project was to evaluate ScAT inflammasome in human cachexia, we prioritized this analysis. As far as we know, this is the first study to show that the NLRP3 pathway is also activated in human cachexia (similarly to the previously demonstrated in rodents).

## Data Availability Statement

The original contributions presented in the study are included in the article/supplementary material. Further inquiries can be directed to the corresponding authors.

## Ethics Statement

The studies involving human participants were reviewed and approved by Faculdade de Medicina, Hospital Universitário and Instituto de Ciências Biomédicas - University of São Paulo. The patients/participants provided their written informed consent to participate in this study.

## Author Contributions

JJ, JO, and MS conceived the project. JJ performed the experiments, with the help of AM, KR, RM, RF, and AP. JJ wrote the manuscript. JO and MS ensured funding, supervised the project, and helped to correct the manuscript. JR-N and EM-N provided technical support. PA, FT, LM, FB, and FF provided the adipose tissue samples. All authors have contributed equally to this work. All authors contributed to the article and approved the submitted version.

## Funding

We thank CAPES (Coordenação de Aperfeiçoamento de Pessoal de Nível Superior) for the scholarship. Financial support was provided by FAPESP 12/50079-0 and 20/7765-6.

## Conflict of Interest

The authors declare that the research was conducted in the absence of any commercial or financial relationships that could be construed as a potential conflict of interest.

## Publisher’s Note

All claims expressed in this article are solely those of the authors and do not necessarily represent those of their affiliated organizations, or those of the publisher, the editors and the reviewers. Any product that may be evaluated in this article, or claim that may be made by its manufacturer, is not guaranteed or endorsed by the publisher.
